# Unreduced Male Gamete Formation in *Cymbidium* and Its Use for Developing Sexual Polyploid Cultivars

**DOI:** 10.3389/fpls.2020.00558

**Published:** 2020-05-15

**Authors:** Rui-Zhen Zeng, Jiao Zhu, Shi-Ying Xu, Guo-Hui Du, He-Rong Guo, Jianjun Chen, Zhi-Sheng Zhang, Li Xie

**Affiliations:** ^1^College of Forestry and Landscape Architecture, South China Agricultural University, Guangzhou, China; ^2^Guangdong Provincial Key Laboratory of Plant Molecular Breeding, South China Agricultural University, Guangzhou, China; ^3^Environmental Horticulture Department, Mid-Florida Research and Education Center, Insititute of Food and Agrocultural Sciences (IFAS), University of Florida, Apopka, FL, United States

**Keywords:** 2*n* gametes, *Cymbidium*, floriculture crops, micropropagation, sexual polyploidization

## Abstract

Polyploidy plays an important role in crop improvement. Polyploid plants, particularly those produced through unreduced gametes (2*n* gametes), show increased organ size, improved buffering capacity for deleterious mutations, and enhanced heterozygosity and heterosis. Induced polyploidy has been widely used for improving floriculture crops, however, there are few reported sexual polyploid plants in the floriculture industry. This study evaluated nine cultivars of *Cymbidium* Swartz and discovered that 2*n* male gametes occurred in this important orchid. Depending on cultivars, 2*n* male gamete formation frequencies varied from 0.15 to 4.03%. Interspecific hybrids generally produced more 2*n* male gametes than traditional cultivars. To generate sexual polyploid plants, seven pairs of crosses were made, which produced five triploid and two tetraploid hybrids. Two triploid hybrids were evaluated for *in vitro* regeneration and growth characteristics. Compared to the diploid parents, the triploids were more easily regenerated through rhizomes or protocorms, and regenerated plants had improved survival rates after transplanting to the greenhouse. Furthermore, the sexual polyploid plants had more compact growth style, produced fragrant flowers, and demonstrated heterosis in plant growth. Through this study, a reliable protocol for selection of appropriate parents for 2*n* gamete production, ploidy level evaluation, *in vitro* culture of polyploid progenies, and development of new polyploid cultivars was established. Our study with *Cymbidium* suggests that the use of 2*n* gametes is a viable approach for improving floriculture crops.

## Introduction

*Cymbidium* Swartz, or boat orchid, is a genus of evergreen flowering plants in the family Orchidaceae. It is mainly native to China and northern Asia and is one of the most important orchids produced commercially as cut flowers and potted flowering plants around the world (Xie et al., 2017). There are 44 recognized species (Griffiths and Huxley, 1992), which have a diploid number of chromosomes of 40. Among them, terrestrial *Cymbidium sinense*, *C. ensifolium*, *C. faberi*, *C. kanran*, and *C. goeringii* are the most popular and economically significant ornamental plants in China. These species have been cultivated since the time of Confucius (551–479 BC) because of their graceful leaves, erect inflorescences, and sweet-scented flowers (Liu et al., 2017). Today, interest in cymbidiums has shifted from cultivars with small flowers to those possessing large and round flowers with long-lasting inflorescences and fragrance and more robust stems and leaves.

Polyploid plants generally show increased organ size and improved tolerance to stressful environmental conditions (Baduel et al., 2018; Pham et al., 2019). Polyploidization has been used to develop hybrids with floral and growth characteristics unobtainable from diploid forms, such as *Platanus acerifolia* (Liu et al., 2007), *Miscanthus* (Głowacka et al., 2010; Miguel and Leonhardt, 2011), *Acacia senegal* (Diallo et al., 2016), *Plectranthus esculgentusare* (Hannweg et al., 2016), and poinsettia (Pan et al., 2019). Ornamental plants are valued for their aesthetic appearance, including plant canopy height and architecture; leaf shape, texture, and thickness; flower form and color (Henny and Chen, 2003). Through polyploidization, ornamental plants with larger and heavier flowers and prolonged flowering period have been developed (Głowacka et al., 2010; Sattler et al., 2016; Marasek-Ciolakowska et al., 2018; Manzoor et al., 2019). Polyploidization also resulted in plants with thicker leaves with increased width-to-length ratio, robust stems, deeper green leaves, more compact growth form (Eeckhaut et al., 2004; Liu et al., 2007; Zhang et al., 2008) as well as delayed flowering time (Väinölä, 2000; Eeckhaut et al., 2004; Vichiato et al., 2014). In fact, many popular cultivars of orchids are polyploid (Zhu et al., 2006; Chen et al., 2009; Liao et al., 2012; Xie et al., 2014). Polyploid orchids usually have more sturdy stems and thicker leaves, larger flowers with more intense color, and rounder conformation (MacLeod, 1947; Kamemoto and Kam, 1980; Griesbach, 1985; Wimber et al., 1987; Watrous and Wimber, 1988; Chen et al., 2009; Miguel and Leonhardt, 2011; Vichiato et al., 2014), thus exhibiting improved ornamental value.

Polyploidy occurs asexually through somatic chromosome doubling and sexually through the formation of unreduced gametes or 2*n* gametes (Ramanna et al., 2003; Gallo et al., 2007; Fakhri et al., 2016) and polyspermy (Tekleyohans and Groß-Hardt, 2019; Toda and Okamoto, 2019). Mitotic chromosome doubling has been achieved using antimitotic agents, such as colchicine and oryzalin. These agents inhibit mitosis during metaphase by interfering with the function of microtubules and lead to the production of polyploid plants. This technique has been intensively used for inducing polyploid cymbidiums (Menninger, 1963; Wimber and Van Cott, 1966; Kim et al., 1997; Wang et al., 2010, 2011; Yin et al., 2010; Ji et al., 2011; Xie et al., 2017). Unreduced gamete is an important pathway of generating polyploid plants and is a driving force behind the formation of polyploids in nature (Otto and Whitton, 2000; Hegarty and Hiscock, 2008; Soltis et al., 2009; Dewitte et al., 2011; Mason and Pires, 2015; Kreiner et al., 2017a). Compared to somatic polyploidization, sexual polyploidization can be of advantageous in plant breeding due to the resultant genetic diversity and heterosis (Ogburia et al., 2002; Ramanna and Jacobsen, 2003; Brownfield and Köhler, 2010; Khan et al., 2010; Dewitte et al., 2011; Younis et al., 2014; Lai et al., 2015). Sexual polyploidization had been used to develop polyploid ornamental plants including lily (Barba-Gonzalez et al., 2004; Zhou et al., 2008; Khan et al., 2010), tulip (Marasek-Ciolakowska et al., 2014), and *Phalaenopsis* (Zhou et al., 2009). However, there has been little available information on 2*n* gamete production in *Cymbidium* and its use in crop improvement. Polyspermy is referred to as an egg cell fertilized by more than one sperm cell, generating viable progeny in flowering plants (Chaudhary et al., 2020). The potential of polyspermy in plant breeding is yet to be exploited (Mao et al., 2020).

The objectives of this study were to explore 2*n* gamete occurrence in cymbidium cultivars and establish a protocol for 2*n* gamete identification, production of polyploid plants through hybridization, and assessment of ornamental value through morphological evaluation. Our effort has resulted in the discovery of 2*n* gamete formation in cymbidium and development a reliable protocol for improving cymbidium via sexual polyploidization.

## Materials and Methods

### Plant Materials

A total of nine cultivars were used in this study (Table 1 and Supplementary Figure S1). Five of them were traditional cultivars of *C. sinense.* The remaining “Dafeng,” “Yunv,” “45–17,” and “45–32” were hybrids. “Dafeng” and “Yunv” were developed from the cross of *C. sinense* with *C. “*Sleeping Beauty” and *C. sinense* with *C.* “King Arthur,” respectively. Whereas “45–17” and “45–32” were developed from interspecific crosses between *C. sinense* and *C. lancifolium.* Plants were grown in black plastic planting bags (2.6 L) filled with a mixture of pine bark (2–4 cm in length) with granite (0.5–1 cm in length) in 3 to 1 ratio based on volume in a shaded greenhouse at the Experimental Farm of South China Agricultural University, Guangzhou, China. Photosynthetically active radiation ranged from 300 to 400 μmol m^–2^ s ^–1^. After plant establishment, 4 g of a slow-release fertilizer (N–P_2_O_5_–K_2_O; 20–20–20) was applied in each planting bag in March and September, respectively each year, and a solution containing 0.1% KH_2_PO_4_ (w/v) was also sprayed every month during the growing season. Plants started flowering in the middle of December, and the flowering lasted for 4 months.

**TABLE 1:** *Cymbidium* cultivars used for evaluation of 2*n* gamete formation.

Species	Cultivar	Description
C. *sinsense*	Qijianbaimo	A traditional cultivar developed through germplasm introduction and enhancement, which has long and dark green leaves, small, white-jade and perfume flower, blooming in February and March, flowering lasted 15–20 days
*C. sinense*	Damo	A traditional cultivar developed through germplasm introduction and enhancement. It has short and thick leaves with distortion in the middle, purplish and perfume flower, blooming in February and March, flowering lasted 15–20 days
*C. sinense*	Hezhihua	A traditional cultivar developed through germplasm introduction and enhancement, which has wide leaves with line art, perfume and reddish-brown flower
*C. sinense*	Xiaoxiang	A traditional cultivar developed through germplasm introduction and enhancement. It has wide and dark green leaves, reddish brown flower with heavy perfume
*C. sinense*	Taipingyang	A traditional non-hybrid cultivar, semi-erect leaves with line art, perfume and purple-red flower.
*C.* ×	Dafeng	A hybrid developed from a cross of *C. sinense* and *C.* × Sleeping Beauty. It has long and semi-erect leaves, large and yellow flower with heavy perfume, blooming in January and February, flowering lasted about 50 days
*C.* ×	Yunv	A hybrid of *C. sinense* and *C.* × King Arthur, long and erect leaves, large and light yellowish-green flower with gently perfume, blooming in January and February, flowering lasted about 50 days
*C.* ×	45–17	A hybrid of *C. sinense* and *C. lancifolium*, light green and graceful leaves, purplish red and perfume flower, blooming in December and January, flowering lasted 40 days
*C.* ×	45–32	A hybrid of *C. sinense* and *C. lancifolium*, light green and graceful leaves, purplish red and perfume flower, blooming in December and January, flowering lasted 40 days

### Determination of Unreduced Male Gametes

To examine the occurrence of unreduced male gametes, pollinia of a blooming flower from different plants were transferred to a slide, crushed and stained with a drop of improved carbolfuchsin solution (Verhoeff, 1912) for 3–5 min or a drop of 4,6-diamidino-2-phenylindole (DAPI) [2 μg.ml^–1^ DAPI, 1% Triton X-100 (v/v), and 1% sucrose (w/v)] in the dark for 10 min. After a cover glass was placed on each slide, the cover glass was squeezed with pencil eraser. The slides were observed under light or UV illumination with ZEISS microscope. Photographs were taken under binocular light microscope with image-forming system. The number of dyads, triads, and tetrads in light vision field of microscope was counted, respectively. Ten vision fields were recorded for each slide, which was regarded as a replicate; each sample replicated three times. The frequency for unreduced male gamete occurrence (F_2__n_) was calculated based on the equation F_2__n_ (%) = (2Dy + Tr)/(2Dy + 3Tr + 4Te) × 100, where Dy, Tr, and Te were the number of dyads, triads, tetrads, respectively. This experiment was carried out for successive 3 years.

### Hybridization

After determination of the frequency of 2*n* gamete formation, healthy plants with blooming flowers were chosen for hybridization, which included “Dafeng” × “Hezhihua,” “Yunv” × “Qijianbaimo,” “Qijianbaimo” × “Damo,” “Yunv” × “Xiaoxiang,” “Yunv” × “Taipingyang,” “45–32” × “45–17,” and “45–32” × “45–32” (Table 2 and Supplementary Table S1). Flower lip and pollinia were removed from a blooming flower of female parent, the stigma was pollinated with pollen from a male parent, i.e., a tooth stick was inserted into male pollinium, and the pollinium was placed into the stigmatic chamber of female flower. A label with date for each cross and their parents was attached to each pollinated flower. The number of pollinated flowers and mature capsules in each cross combination were recorded individually, and the success rate of crosses was calculated.

**TABLE 2:** Ploidy evaluation of *Cymbidium* hybrids resulted from different crosses.

Cross combination (♀ × ♂)	Total no. of hybrid produced	No. of diploid	No. of triploid (Occurrence frequency of triploid/%)	No. of tetraploid (Occurrence frequency of tetraploid/%)
“Dafeng” × “Hezhihua”	830	829	1 (0.12)	0
“Yunv” × “Qijianbaimo”	279	279	0	0
“Qijianbaimo” × “Damo”	200	200	0	0
“Yunv” × “Xiaoxiang”	300	299	1 (0.33)	0
“Yunv” × “Taipingyang”	532	529	1 (0.18)	1 (0.18)
“45–32” × “45–17”	58	56	2 (3.45)	0
“45–32” × “45–32”	54	53	0	1 (1.85)

### Hybrid Seed Germination and Propagation

Mature capsules that resulted from the crosses were collected. After removing the fruit stalk and remaining column, the capsules were washed in running tap water, surface sterilized with a 75% (v/v) ethanol solution for 8–10 min and rinsed with sterilized distilled water three times. The sterilized capsules were longitudinally cut, released seeds were cultured on half-strength MS (Murashige and Skoog, 1962) medium supplemented with 0.5 mg⋅L^–1^ 6-BA (6-benzylaminopurine), 0.2 mg⋅L^–1^ NAA (naphthaleneacetic acid), 30 g⋅L^–1^ sucrose, 7.5 g⋅L^–1^ agar, 100 ml⋅L^–1^ coconut water (CW), and 0.5 g⋅L^–1^ active carbon (AC). The pH of the medium was adjusted to 5.8 with 1 *M* KOH or 1 *M* HCl prior to autoclaving for 20 min at 121°C. Seed germination took place in a controlled environment in the dark at 26°C ± 1°C. Seeds produced small, round, and green protocorm or rhizome.

A small piece of rhizome produced from individual seeds was cultured on half-strength MS medium supplemented with 1.0 mg⋅L^–1^ 6-BA, 0.2 mg⋅L^–1^ NAA, 30 g⋅L^–1^ sucrose, 7.5 g⋅L^–1^ agar, and 0.5 g⋅L^–1^ AC. They were maintained in a culture room under a light intensity of 40 μmol⋅m^–2^⋅s^–1^ for 12 h and a temperature of 26°C ± 1°C. In order to produce a large number of propagules from each seed, rhizomes derived from the seed were sub-cultured five times, 50 days each. Subsequently, the propagules were cultured on half-strength MS medium containing 2.0 mg⋅L^–1^ 6-BA, 0.2 mg⋅L^–1^ NAA, 30 g⋅L^–1^ sucrose, and 7.5 g⋅L^–1^ agar, and 0.5 g⋅L^–1^ AC for shoot induction. When the shoots reached 4 cm in height, they were transferred to half-strength MS medium supplemented with 0.2 mg⋅L^–1^ 6-BA, 0.5 mg⋅L^–1^ NAA, 20 g⋅L^–1^ sucrose, 7.5 g⋅L^–1^ agar, and 0.5 g⋅L^–1^ AC for rooting. Plantlets derived from each seed of a cross were a considered to be a breeding line. Based on morphological characteristics, mainly increased stem diameter and leaf size and thickness as well as shorter and thicker roots of the plantlets, putative polyploid lines were selected.

### Chromosome Counts

A total of 30 plantlets from each putative polyploid line were randomly selected for counting chromosome numbers of root tip using the squash method (Wang et al., 2010). Briefly, about 0.2 cm of the root-tip was removed from a seedling and pretreated in 2.0 mM 8-hydroxyquinoline solution at 18°C in the dark for 4 h. The root-tips were fixed in Carnoy’s solution [a 3:1 (v/v) ratio of 95% (v/v) ethanol and glacial acetic acid] at 4°C for 24 h. After washing with distilled water three times, they were subjected to the acidolysis in 1 *M* HCl solution at 60°C for 12 min. After being soaked in distilled water for 30 min, the fixed apex was stained with improved carbolfuchsin (Verhoeff, 1912) by squeezing them with a needle on a slide. Debris and excess stain were removed and the sample was covered with a cover-slip and observed at 100 × magnification using a photomicroscope (Olympus-IX71, Japan), a digital camera system (Nikon) were used for photography. For each plantlet, at least 20 cells were observed. If more than 90% of the cells had a constant chromosome number, the chromosome number of the plantlet was confirmed.

### Flow Cytometric Analysis

The relative DNA content of the polyploid plantlets and their counterparts was measured using flow cytometry (Cui et al., 2009; Corneillie et al., 2019) with a slight modification. Approximately 0.5 cm^2^ of leaves was collected and chopped into small pieces with a sharp razor blade in a 55 mm plastic Petri dish containing 0.4 mL of Partec HR-A (Partec GmbH, Münster, Germany), followed by adding 1.6 mL of Partec HR-B with DAPI as DNA staining agent. The nuclei suspension was subsequently filtered through a disposable filter with 30 μm mesh size (Partec, Celltrics, Germany), and kept on ice in the dark for 3–5 min. Samples were analyzed using a Partec PA-II flow cytometer. Collected data were analyzed simultaneously using CyView 8.5 software (Partec GmbH, Münster, Germany) and presented as DNA histograms. Five plants from each line were analyzed. Based on the peaks obtained in the histograms, ploidy levels were estimated as diploid, triploid, and tetraploid.

### Plant Production

To produce mature plants, identified polyploid plantlets, along with their diploid counterparts at a height of 8 cm were rinsed with tap water and briefly air dried, and then transplanted into small black plastic planting bags (100 mL) filled with a substrate comprised of small pine bark (1 cm in length) and peat in a 3:1 ratio based on volume, one plantlet per bag. Plants were grown in a shaded greenhouse under a light intensity of 120 μmol⋅m^–2^⋅s^–1^, temperature ranging from 15°C to 30°C, and relative humidity varying from 70 to 80%. The plants were fertigated with a Hyponex (N–P_2_O_5_–K_2_O; 20–20–20) solution every 10 d. After 6 months, they were transplanted into large black plastic planting bags (2.6 L) filled with a substrate comprised of pine bark (2–4 cm in length) and granite (0.5–1 cm in length) in a 3:1 ratio based on volume and cultivated in another shaded greenhouse under a light intensity of 300–400 μmol m^–2^ s ^–1^, 3–4 g of a slow-release fertilizer (N–P_2_O_5_–K_2_O; 20–20–20) was applied to each planting bag on March and September, respectively, and a solution containing 0.1% KH_2_PO_4_ (w/v) was sprayed every month during the growing season.

### Evaluation of Ornamental Characteristics

Two polyploid cultivars, Yutao and Huanghe along with their diploid counterparts XY and DH were evaluated for their ornamental characteristics. Ten plants were randomly selected from each cultivar. Main morphological characteristics were recorded according to guidelines for testing the distinctness, uniformity, and stability of *Cymbidium* (International Union for the Protection of New Varieties of Plants [UPOV], 1999). Leaf and flower numbers were recorded, the diameter of pseudobulb and inflorescence stalk and the thickness of leaves, sepal and petal were measured using an automatic caliper, and other traits, such as plant height and leaf length and width, were measured using a ruler.

### Assessment of Regeneration Capacity

Two triploid cultivars: Yutao and Huanghe and a diploid DH were used for evaluating regeneration capacity. Shoot tips were excised from 8 cm plantlets, cut into 1 mm in length, and cultured in glass culture vessels (height: 10 cm, diameter: 6.5 cm, and volume: 330 mL) containing MS medium. The medium was supplemented with 1.0 mg⋅L^–1^ 6-BA, 0.2 mg⋅L^–1^ NAA, 50 ml L^–1^ CW, 30 g⋅L^–1^ sucrose, 7⋅g L^–1^ agar, and 0.5 g⋅L^–1^ AC with a pH of 5.8. The cultures were maintained in darkness at 26°C for 40 days. There were three explants per culture vessel, 30 vessels per treatment. Rhizome induction rates were calculated using the formula: induction rate (%) = (N_rhi_/3) × 100, where N_rhi_ represented the number of explants forming rhizomes in each culture vessel.

To determine the proliferation of rhizomes, about 2 g of rhizome, each 1 cm in length were inoculated in a culture vessel containing 30 mL MS medium supplemented with 1.0 mg⋅L^–1^ 6-BA, 0.2 mg⋅L^–1^ NAA, 30 g⋅L^–1^ sucrose, 7 g⋅L^–1^ agar, and 0.3 g⋅L^–1^ AC with a pH at 5.8. There were 15 culture vessels per cultivar, which were cultured under a 12 h photoperiod with a light intensity of 20 μmol m^–2^s ^–1^ at 26°C for 40 days. The fresh weight of rhizomes in each vessel was measured using an electronic balance. The proliferation coefficient was calculated based on the weight of the rhizomes in a vessel after 40 days of culture divided by the initial weight of the rhizomes in the same vessel.

To evaluate shoot regeneration from rhizomes, 10 rhizomes, each in a length of about 1 cm were inoculated in a culture vessel containing 30 ml MS medium supplemented with 1.0 mg⋅L^–1^ 6-BA, 0.2 mg⋅L^–1^ NAA, 30 g L^–1^ sucrose, 7 g⋅L^–1^ agar, and 0.02 g⋅L^–1^ AC with a pH at 5.8. There were 15 vessels per cultivar, and they were maintained under a 12 h photoperiod with a light intensity of 40 μmol m^–2^s^–1^ at 26°C for 40 days. The numbers of shoots regenerated from rhizomes per vessel were recorded. The shoot regeneration rate was calculated by the formula: shoot regeneration rate (%) = (the number of rhizomes regenerating shoots in a vessel/10) × 100.

The regenerated shoots were cultured on half-strength MS medium containing 0.1 mg⋅L^–1^ 6-BA, 0.5 mg⋅L^–1^ NAA, 20 g⋅L^–1^ sucrose, and 0.5 g⋅L^–1^ AC under a light intensity of 40 μmol m^–2^s ^–1^ at 26°C for 50 days. Plantlets at a height of 8 cm were washed with tap water and transplanted to the aforementioned substrate. The plants were grown in a shaded greenhouse as described above for producing mature plants. A total of 120 plantlets were transplanted per cultivar, which were arranged as randomized complete block design with three blocks, 40 plantlets per block. After 60 days, the number of surviving seedlings were recorded using the formula: survival rate (%) = (the number of seedling survived in a replicate/40) × 100.

### Statistical Analysis

Collected data were subjected to analysis of variance (ANOVA) using statistical program SPSS 22.0 (IBM Corporation, Somers, NY). When significance occurred, means were separated by Duncan’s multiple range test at *P* < 0.05 level.

## Results

### Occurrence in Unreduced Male Gamete

**FIGURE 1:**
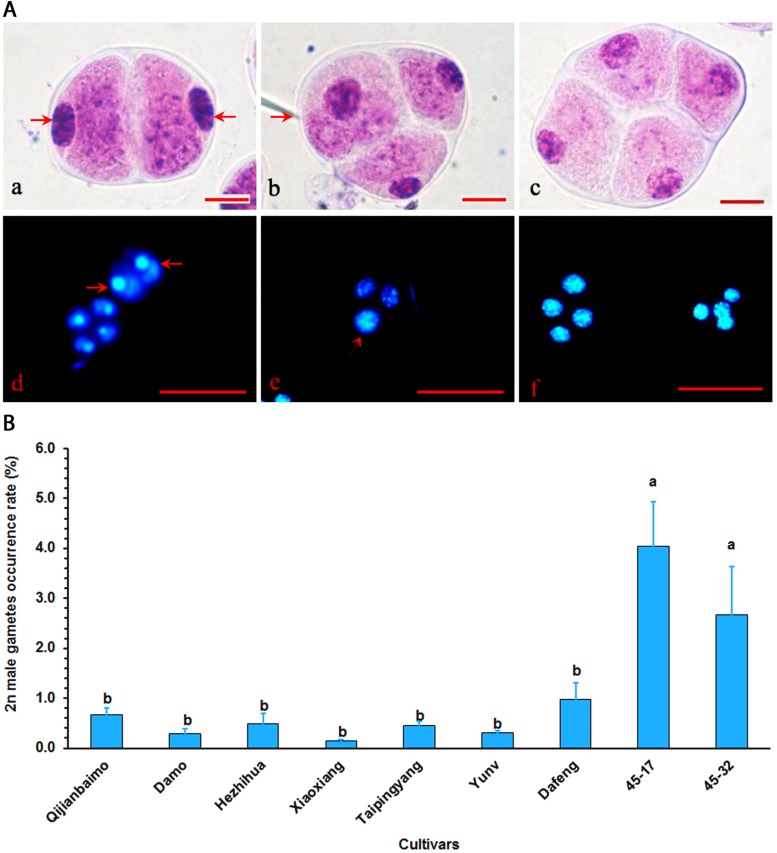
Analysis of unreduced male gametes in *Cymbidium* cultivars using the squash method. **(A)** Mature pollens were stained with carbolfuchsin, and dyad (a), triad (b), and tetrad (c) gametes were identified by the squash method. Bar = 10 μm. The dyad with 2*n* gametes (arrow) (d), triads with one unreduced gamete (arrow) and two reduced gametes (e), and tetrad with four reduced gametes (f) were stained with 4’,6-diamidino-2-phenylindole (DAPI). Bar = 50 μm. **(B)** 2*n* gamete formation frequencies (means of 3-year data, Supplementary Table S1) among nine cultivars, bars represent standard error (*n* = 30). Different letters on the top of bars indicate significant cultivar difference in 2*n* gamete formation frequencies analyzed by Duncan’s multiple range test at *P* < 0.05 level.

All nine parental cultivars produced unreduced male gametes, which were either dyad or triad (Figure 1A and Supplementary Table S1). Cultivars, however, varied significantly in 2*n* male gamete formation frequency, ranging from 0.15% in “Xiaoxiang” to 4.03% in “47–17” (Figure 1B). Generally, hybrid, particularly those interspecific ones had higher 2*n* gamete formation frequencies than traditional cultivars.

### Hybrid Seeds and *in vitro* Propagation

Seven pairs of crosses were made using the nine parental cultivars. All the crosses were compatible, resulting in 100% success rate (Supplementary Table S2). The resultant capsules matured at different times, varying from 210 days for the cross of “Yunv” × “Taipingyang” to 310 days for the cross of “Dafeng” × “Hezhihua.” The numbers of hybrid seeds produced from the crosses differed greatly, and the cross of “Dafeng” × “Hezhihua” produced more hybrid seeds than the cross of “45–32” × “45–32” (Table 2).

To ensure appropriate germination of the hybrid seeds, they were propagated through *in vitro* culture (Supplementary Figure S2). The seeds required at least 150 days to germinate and germination rate differed from 30% to 90% (Supplementary Table S2). The germination rates of seeds derived from crosses of “Dafeng” × “Hezhihua,” “Yunv” × “Taipingyang,” and “Qijianbaimo” × “Damo” were estimated to be 70%, which were higher than those derived from interspecific crosses “45–32” × “45–17” or self-cross of “45–32” which were only 30%.

### Evaluation of Ploidy Levels of Hybrids

**FIGURE 2:**
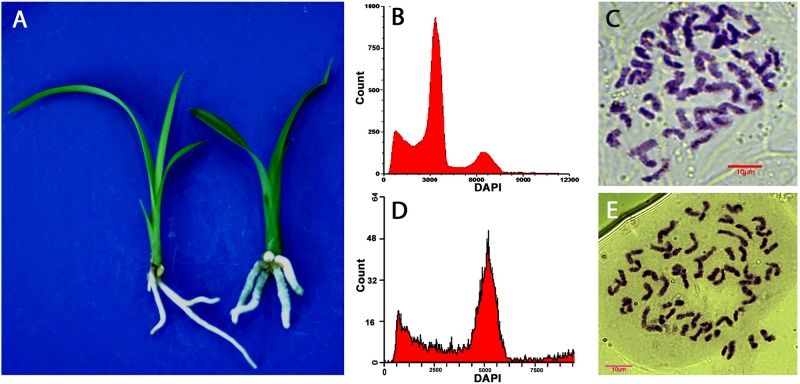
Evaluation of ploidy levels of *Cymbidium* hybrids of “DH” and “Huanghe” resulted from cross of “Dafeng” × “Hezhihua.” **(A)** Morphology of “DH” (left) and “Huanghe” (right). **(B)** Flow cytometric DNA histograms of “DH” and **(C)** chromosome numbers of root tip cell of “DH” (2*n* = 2*x* = 40). **(D)** Flow cytometric DNA histograms of “Huanghe” and **(E)** chromosome numbers of root tip cell of “Huanghe” (2*n* = 3*x* = 60).

**FIGURE 3:**
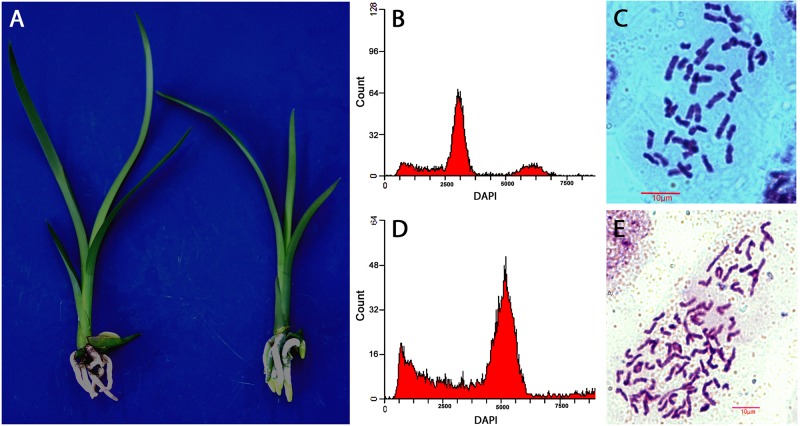
Evaluation of ploidy levels of *Cymbidium* hybrids of “XY” and “Yutao” developed from cross of “Yunv” × “Xiaoxiang.” **(A)** Morphology of “YX” (left) and “Yutao” (right). **(B)** Flow cytometric DNA histograms of “YX” and **(C)** chromosome numbers of root tip cell of “YX” (2*n* = 2*x* = 40). **(D)** Flow cytometric DNA histograms of “Yutao” and **(E)** chromosome numbers of root tip cell of “Yutao” (2*n* = 3*x* = 60).

Ploidy levels of *in vitro* propagated seedlings were examined through morphological evaluation, chromosome counting, and flow cytometric analysis. The results showed that majority of the hybrids were diploid, but few were triploid or tetraploid (Table 2). The cross of “Dafeng” × “Hezhihua” produced a triploid plant “Huanghe,” which had 2*n* = 3*x* = 60 compared to its diploid counterpart of “DH” with 2*n* = 2*x* = 40 (Figure 2). Flow cytometric analysis also confirmed that “Huanghe” was a triploid. Morphologically, “Huanghe” had shorter but thicker roots and was more robust, its leaves were darker green compared with “DH” (Figure 2). A triploid called “Yutao” was also identified from the cross of “Yunv” × “Xiaoxiang” (Figure 3). Both chromosome counting and flow cytometric analysis confirmed that it was a triploid with 2*n* = 3*x* = 60. One triploid and one tetraploid were found from the cross of “Yunv” × “Taipingyang” (Supplementary Figure S3). Two triploid plants were identified from the cross of “45–32” × “45–17” (Supplementary Figure S4). Additionally, one tetraploid was found from self-pollinated progenies called “45–32” (Supplementary Figure S5). The chromosome counting results were further confirmed by flow cytometric analysis and morphological evaluation.

### Ornamental Traits of Polyploid Plants

**FIGURE 4:**
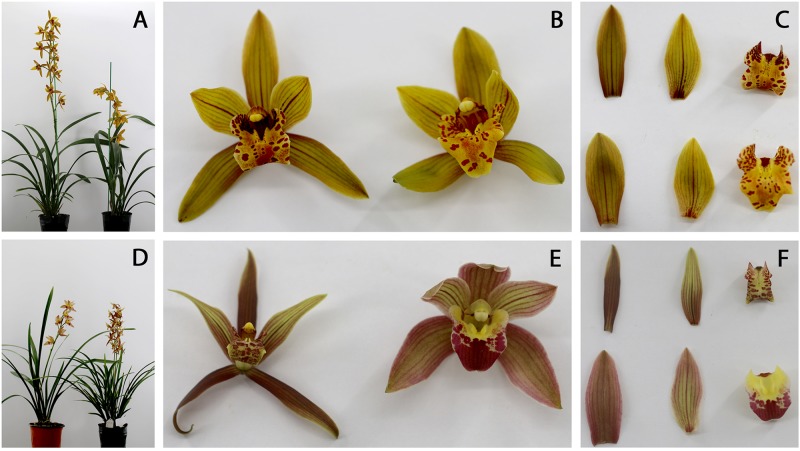
Morphological characteristics of polyploid *Cymbidium* cultivars with their diploid counterparts. **(A)** Mature plants of “DH” (left) and “Huanghe” (right). **(B)** Flowers of “DH” (left) and “Huanghe” (right). **(C)** Sepals (left), petals (middle), and lips (right) of “DH” (above) and “Huanghe” (below). **(D)** Mature plants of “XY” (left) and “Yutao” (right); **(E)** Flowers of “YX” (left) and “Yutao” (right); **(F)** Sepals (left), petals (middle), and lips (right) of “YX” (above) and “Yutao” (below).

Ornamental characteristics were comparatively evaluated between selected triploid and the diploid counterpart. Results showed sexual polyploidization caused significant changes in morphological traits. For example, plant canopy width and lip width significantly increased, but flower numbers and peduncle length noticeably decreased in triploid “Huanghe” compared to the diploid “DH,” but other parameters were not significantly altered (Table 3 and Figures 4A–C). On the other hand, canopy height; pseudobulb diameter; leaf length, width, and thickness; peduncle thickness; sepal length, width, and thickness; petal width and thickness as well as lip width of triploid “Yutao” were significantly greater than the diploid “XY” (Table 3 and Figures 4D–F). Aesthetically, sexual polyploidization increased the width and thickness of sepal, petal, and lip. Flowers became much rounder in shape and produced fragrance, thus ornamental value was greatly improved (Figures 4B,E). Additionally, triploids “Yutao” and “Huanghe” were much sturdier and more robust compared with their counterparts (Figures 4A,D). However, there were no significant differences in the time of flowering between triploids and diploid counterparts.

**TABLE 3:** The effect of sexual polyploidization on morphological characteristics of *Cymbidium* cultivars.

Character	“Huanghe”	“DH”	“Yutao”	“YX”
Plant height (cm)	35.0 ± 4.62a	41.8 ± 0.29a	51.2 ± 1.70a	37.1 ± 3.43b
Plant width (cm)	76.0 ± 6.43a	53.7 ± 3.75b	50.9 ± 2.72a	46.7 ± 4.50a
No. of leaf	6.3 ± 0.17a	5.3 ± 0.33a	6.7 ± 0.38a	5.7 ± 0.33a
Pseudobulb diameter (cm)	2.8 ± 0.53a	3.1 ± 0.01a	2.8 ± 0.06a	1.8 ± 0.03b
Leaf Length (cm)	64.9 ± 5.80a	58.5 ± 6.09a	60.3 ± 1.16a	47.6 ± 2.00b
Leaf width (cm)	2.2 ± 0.15a	2.6 ± 0.09a	2.2 ± 0.05a	1.2 ± 0.03b
Leaf thickness (mm)	0.8 ± 0.02a	0.7 ± 0.04a	0.7 ± 0.02a	0.6 ± 0.01b
No. of flowers	7.0 ± 0.00a	10.3 ± 0.69b	7.5 ± 0.31a	6.5 ± 0.29a
Peduncle length (cm)	54.1 ± 0.22a	72.8 ± 4.29b	49.8 ± 2.96a	45.6 ± 0.07a
Peduncle thickness (cm)	0.7 ± 0.02a	0.7 ± 0.04a	0.7 ± 0.02a	0.4 ± 0.00b
Flower width (cm)	7.0 ± 0.36a	7.3 ± 0.11a	4.9 ± 0.19a	5.7 ± 0.38a
Flower Length (cm)	7.6 ± 0.35a	7.8 ± 0.23a	5.3 ± 0.07a	6.1 ± 0.32a
Sepal length (cm)	4.6 ± 0.02a	4.5 ± 0.07a	4.0 ± 0.06a	4.2 ± 0.04b
Sepal width (cm)	1.9 ± 0.06a	1.6 ± 0.10a	1.7 ± 0.02a	0.8 ± 0.00b
Sepal thickness (mm)	0.8 ± 0.02a	0.7 ± 0.03a	0.9 ± 0.01a	0.5 ± 0.01b
Petal length (cm)	4.1 ± 0.02a	4.1 ± 0.11a	3.5 ± 0.04a	3.5 ± 0.03a
Petal width (cm)	2.1 ± 0.17a	1.8 ± 0.15a	1.4 ± 0.02a	0.9 ± 0.02b
Petal thickness (mm)	0.8 ± 0.01a	0.8 ± 0.05a	0.8 ± 0.01a	0.5 ± 0.01b
Lip length (cm)	3.8 ± 0.01a	3.8 ± 0.04a	3.0 ± 0.03a	3.1 ± 0.04a
Lip width (cm)	3.6 ± 0.07a	2.9 ± 0.02b	2.5 ± 0.03a	1.6 ± 0.04b

*“Yutao” (3x) and “YX” (2x) come from the cross of “Yunv” × “Xiaoxiang,” “Huanghe” (3x) and “DH” (2x) come from the cross of “Dafeng” × “Hezhihua.” Values represent mean ± standard error (n = 3). Different letters in the same row of “Yutao” and “YX” or “Huanghe” and “DH” indicate significant difference based on Duncan’s multiple range test at P < 0.05 level.*

### Regeneration of Polyploid Plants

**FIGURE 5:**
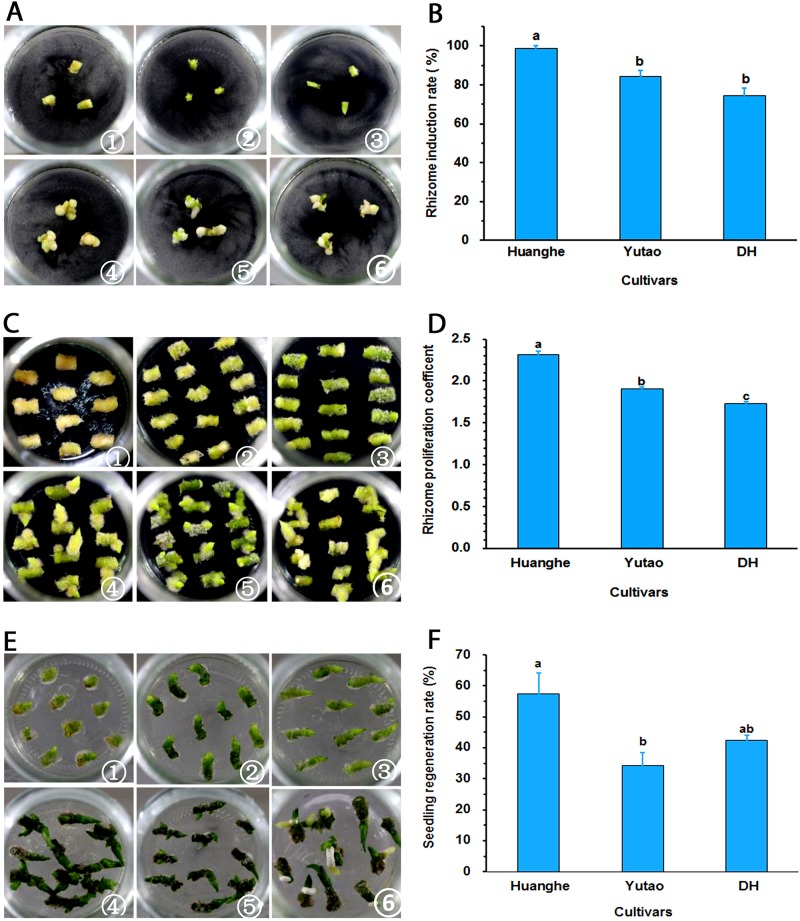
Regeneration ability of polyploid *Cymbidium* cultivars developed from sexual polyploidization. **(A)** Induction of rhizomes from shoot tips: ① Shoot tip of “Huanghe” on day one; ② Shoot tip of “Yutao” on day one; ③ Shoot tip of “DH” on day one; ④ Shoot tip of “Huanghe” on day 40; ⑤. Shoot tip of “Yutao” on day 40; ⑥ Shoot tip of “DH” on day 40. **(B)** Rhizome induction frequencies (%) of the three cultivars. **(C)** Rhizome proliferation: ① Rhizomes of “Huanghe” on day one; ② Rhizomes of “Yutao” on day one; ③ Rhizomes of “DH” on day one; ④ Rhizomes of “Huanghe” on day 40; ⑤ Rhizomes of “Yutao” on day 40; ⑥ Rhizomes of “DH” on day 40. **(D)** Rhizome proliferation coefficient of three cultivars. **(E)** Shoot regeneration from rhizomes: ① Shoot induction of “Huanghe” on day one; ② shoot induction of “Yutao” on day one; ③ shoot induction of “DH” on day one; ④ Shoot induction of “Huanghe” on day 40; ⑤ Shoot induction of “Yutao” on day 40; ⑥ Shoot induction of “DH” on day 40. **(F)** Shoot production rate of three cultivars from cultured rhizomes. Bars represent standard error, and different letters on the top of bars indicate significant cultivar difference for individual traits analyzed by Duncan’s multiple range test at *P* < 0.05 level.

**FIGURE 6:**
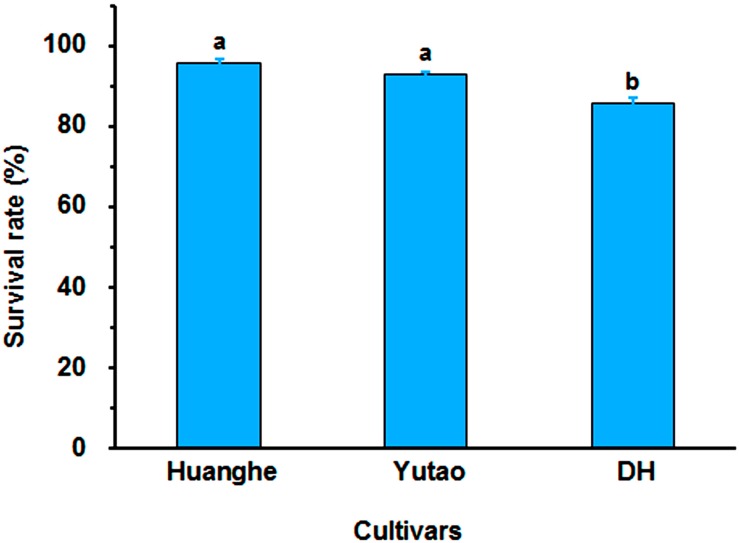
The survival rate of “Huanghe,” “Yutao,” and “DH” plantlets after transplanting into soilless substrate grown in a shaded greenhouse. Bars represent standard error, and different letters on the top of bars indicate significant cultivar difference in survival rates analyzed by Duncan’s multiple range test at *P* < 0.05 level.

Regeneration capacity of polyploid plants was evaluated *in vitro*. The rhizome induction rate of triploid “Huanghe” were significantly higher than triploid “Yutao” and diploid “DH” (Figures 5A,B). The rhizome proliferation coefficient of “Huanghe” was the highest compared to “Yutao” and “DH,” but “Yutao” was significantly higher than “DH” (Figures 5C,D). There was significant difference in shoot regeneration between “Huanghe” and “Yutao,” but the regeneration rate between “Yutao” and “DH” was not statistically significant (Figures 5E,F). However, seedling survival rates of plantlets grown in a shade greenhouse differed significantly. Triploid “Huanghe” and “Yutao” had 96% survival rate compared to 84% of “DH” (Figure 6).

## Discussion

*Cymbidium* is known as the King of Orchids due to its fragrant flowers and multitudes of colors which can remain in bloom for up to 3 months. To improve its ornamental value, many hybrids have been developed from the original 44 species (Obara-Okeyo and Kako, 1998). Additionally, polyploid cymbidiums have been explored asexually through somatic cell chromosome doubling (Wimber and Van Cott, 1966; Kim et al., 1997; Yin et al., 2010; Ji et al., 2011; Wang et al., 2011; Xie et al., 2017). However, sexual polyploidization through unreduced gametes has not been reported in *Cymbidium*. Sexual polyploidization through 2*n* gametes can be of immense significance because it can combine genetic effects of polyploidy with meiotic recombination and sexual hybridization and produce tremendous genetic variation and heterosis (Ogburia et al., 2002; Ramanna and Jacobsen, 2003; Brownfield and Köhler, 2010; Khan et al., 2010; Lai et al., 2015). The occurrence of 2*n* gametes has been reported in some orchids, such as *Calanthe veratrifolia* Lindl., *Plocoglottis javanica* B., and *Spathoglottis plicata* Bl. (Teoh, 1984), but not in *Cymbidium*. The present study as the first to document 2*n* gamete occurrence in *Cymbidium* and production of polyploid cymbidiums sexually. Furthermore, this study established a reliable protocol for 2*n* gamete identification, polyploid plant generation through hybridization, *in vitro* culture of polyploid plants, and evaluation of polyploid plants for new cultivar development. Using this protocol, five triploid and two tetraploid plants were developed, and some evaluated triploids exhibited heterosis in plant growth and improved ornamental value.

The first step of this protocol is to identify cultivars that have potential to produce 2*n* gametes. This was a challenging task as there had been no report of 2*n* gamete formation in *Cymbidium*. In theory, however, the occurrence of 2*n* gametes in a natural plant population is largely due to the dysfunction of meiosis, therefore, all plants propagated sexually could produce 2*n* gametes. Additionally, 2*n* gametes have negative effects on male fitness in diploid population (Kreiner et al., 2017b); thus, the frequency of 2*n* gamete formation could be relatively higher in asexual species because of the existence of an alternative propagation route and also in interspecific or intergeneric hybrids due to the instability of chromosome pairing. Cymbidiums can be propagated by seeds and division. As a result, we selected seven traditional cultivars and two interspecific hybrids “45–17” and “45–32” for evaluation. Common methods for 2*n* gamete identification include microscopic observation of pollen during maturation and flow cytometric analysis (Kreiner et al., 2017b). Using the two methods, we found that the frequency of 2*n* male gamete formation in traditional cultivars ranged from 0.15 to 1.0% but 2.5–4.03% in interspecific hybrids (Figure 1). “47–17” as an interspecific hybrid selected from a cross between *C. sinense* and *C. lancifolium* had a frequency of 4.03%, while “Xiaoxiang” was a traditional variety had a frequency of 0.15%. These 2n gamete formation frequencies are comparable to those reported in 60 populations across 24 species of Brassicaceae where most individuals produced less than 2% of 2*n* male gametes (Kreiner et al., 2017b). Our results clearly indicated that interspecific hybrids produced more 2*n* gametes than traditional cultivars, which concur with several reports from other crops (Ramsey and Schemske, 1998; Fakhri et al., 2016; Kreiner et al., 2017b; Liu et al., 2017). Common pathways for 2*n* gamete formation include first division restriction (FDR) and second division restriction (SDR) (Brownfield and Köhler, 2010). In the FDR, 2*n* gametes are produced from a direct equational division of univalent chromosomes at anaphase I or called pseudo-homotypic division. Whereas in the SDR, 2*n* gametes are resulted from the omission of the second meiotic division following chromosome doubling after anaphase I (Peloquin et al., 2008). The exact mechanisms underlying the formation of dyads and triads in this study (Figure 1A) have not been well elucidated at this time. Our preliminary data suggested that the dyads could be resulted from either FDR or SDR depending on cultivars. The triads could be caused by cell plate abnormality. Further investigation is needed to confirm the propositions.

The next step of this protocol is to produce sexual polyploid plants through hybridization. The occurrence of polyploid progenies could heavily rely on parental plants used for hybridization. We assumed that parents with high 2*n* gamete formation frequencies should produce more polyploid progenies. Meanwhile, polyploid progenies should have desirable ornamental characteristics, which are also dependent on parental combinations. Considering these two factors, we made crosses outlined in Table 2. More hybrid seeds were produced from crosses using traditional cultivars as parents compared to the cross of two interspecific hybrid and the self-cross of “45–32.”

Ensuring maximum germination of hybrid seeds produced from each cross and accurately identifying polyploid individuals from the progenies are critically important for polyploidy breeding. In this step, we propagated seeds *in vitro* to ensure that seed germination took place in appropriate conditions. Geminated seeds were then *in vitro* propagated through rhizome to increase plantlet numbers for ploidy identification and for greenhouse production. Methods used for the ploidy discrimination include squash root tip cells (Lai et al., 2015), guard cell measurements (Miguel and Leonhardt, 2011), genomic *in situ* hybridization (Liu et al., 2017), and flow cytometric analysis (Khan et al., 2010). Our results indicated that the squash method, along with flow cytometry, accurately identified ploidy levels of *Cymbidium* hybrids. Furthermore, we found that seedlings of polyploid progenies had distinguishable morphological characteristics compared to diploid counterparts. Roots of polyploid seedlings were shorter and thicker with much greater diameter in contrast to the roots of diploid seedlings. Additionally, stem diameter and leaves were also thicker (Figures 2A, 3A and Supplementary Figures S3D,G, S4D,G, S5D). Such morphological characteristics substantially assisted in the identification of sexual polyploid individuals from each cross population.

Results from the ploidy evaluation showed that our assumption was partially correct. The hybridization of “45–32” with “45–17,” both had higher 2*n* gamete formation frequencies, produced two triploid plants (Table 2 and Supplementary Figure S4). Additionally, a self-pollination of “45–32” produced one tetraploid (Table 2 and Supplementary Figure S5), suggesting that “45–32” might produce 2*n* eggs. However, the cross between “Yunv” and “Xiaoxiang,” two parents with rather low frequencies of 2*n* gamete formation, produced one triploid progeny (Table 2 and Figure 3). The cross between “Yunv” and “Taipingyang” produced one triploid and one tetraploid (Table 2 and Supplementary Figure S3), which also suggest that “Yunv” could produce 2*n* eggs, whereas, no polyploid progenies were produced from the cross of “Yunv” and “Qijianbaimo” as well as the cross between “Qijianbaimo” and “Damo.” Nevertheless, it appears that parents with high frequencies of 2*n* gamete formation, particularly those interspecific hybrids, have a higher probability to produce polyploid progenies. On the other hand, polyploid progenies could also be produced by parents with relatively low 2*n* male gamete formation frequencies.

The identified polyploid individuals were propagated through *in vitro* culture, which served dual purposes: One was to produce enough plant materials for phenotypic evaluation of ornamental value. The other was to establish reliable methods for increased propagation of the desirable progenies for commercial production. In breeding of field crops, triploid plants are usually sterile due to unbalanced meiotic chromosome segregation and endosperm imbalance (Köhler et al., 2010; Wang et al., 2017). In breeding of ornamental crops, however, a key objective is to improve aesthetic value, not for seed production. Desirable triploids can be effectively propagated asexually using tissue culture, to immediately increase the number of plants for commercial production. Thus, micropropagation plays an important role in the development ornamental plant industry (Chen and Henny, 2008). Micropropagation has been the main method of propagating orchids (Chugh et al., 2009). Somatic polyploidization has been shown to significantly reduce regeneration ability of orchids (Xie et al., 2017; Pham et al., 2019). For example, colchicine induced triploid *Cymbidium hybridum* plants had decreased protocorm-like body (rhizome) proliferation and shoot regeneration (Xie et al., 2017). However, the present study showed that rhizome induction rate and proliferation coefficient, shoot regeneration, and seedling survival rates of triploid “Huanghe” and “Yutao” were either significantly higher than or equal to those of diploid “DH” (Figures 5, 6), suggesting that sexual polyploidization actually improved micropropagation efficiency in *Cymbidium*. The cause of the improved regeneration efficiency is unclear, but it could be implicated by at least two factors. One is the genetic background of two female parents “Dafeng” and “Yunv” used for developing the triploid hybrids “Huanghe” and “Yutao,” respectively. Both “Dafeng” and “Yutao” are relatively easy for regeneration. The other factor could be the meiotic recombination and sexual hybridization resultant genetic epistasis, which requires further investigation.

The final step of this established protocol is to evaluate the performance of sexual polyploidized progenies for developing new cultivars. Morphological novelty, mechanical robustness, increased plant growth, and improved tolerance to stressful factors are highly sought-after traits in ornamental plant breeding. Polyploidization generally increases plant organs, called gigas effect (Sattler et al., 2016). In the genus *Dendrobium*, somatic polyploidization resulted in increased petal and/or sepal sizes and leaf thickness, prolonged durability of flowering but decreased growth rates and flower numbers per pseudobulb (Chaicharoen and Saejew, 1981; McConnell and Kamemoto, 1993; Ketsa et al., 2001; Vichiato et al., 2014). Our results showed that sexual polyploidization significantly increased plant height, pseudobulb diameter, leaf length, width, and thickness, peduncle thickness, sepal width and thickness, petal width and thickness, and lip width of triploid “Yutao” compared to diploid “YX” (Table 3). On the other hand, morphological characteristics of triploid “Huanghe” only showed significant increase in plant width and lip width but decrease in flower numbers and peduncle length compared to diploid “DH” (Table 3). Additionally, both “Yutao” and “Huanghe” had rounder shaped flowers (Figures 4B,E) with much more robustness compared to respective diploid counterparts. Furthermore, triploid “Yutao” grew much faster, exhibiting heterosis. On the contrary, the growth of triploid “Huanghe” was largely comparable to diploid “DH,” exhibiting limited heterosis. Our speculation is that 2*n* gametes produced from parent “Xiaoxiang” could be resulted from the process of FDR, whereas 2*n* gametes from parent “Hezhihua” might be produced through the SDR. In the process of FDR, meiosis I fails to occur, i.e., there are no chromosome pairing and recombination; instead, chromosomes undergo directly to meiosis II, an equational division. Parental heterozygosity and epistasis are fully retained (De Storme and Geelen, 2013). As a result, polyploid hybrids derived from FDR 2*n* gametes possess great genetic diversity and heterosis. Thus, “Yutao” showed increased heterosis in plant growth. On the other hand, chromosomes in SDR have normal pairing and recombination, i.e., normal meiosis I, but an omission of meiosis II. 2*n* gametes resulted from this process generally have reduced level of heterozygosity and shows a substantial loss of parental epistasis (Peloquin et al., 2008). Therefore, “Huanghe” showed less heterosis, but it did show improved efficiency in regeneration. Further research is warranted to confirm these speculations, specifically morphological characteristics of triploid plants produced through sexual polyploidization should be compared with those autoploid plants. Nevertheless, compared to the diploid parents, triploid cultivars produced from this study show improved ornamental value, increased regeneration capacity during *in vitro* culture or enhanced growth. In contrast to these results, somatic polyploidization usually shows reduced regeneration ability and decreased plant growth rates (Chaicharoen and Saejew, 1981; Vichiato et al., 2007). Our results with cymbidium provide additional evidence supporting the advantages of sexual polyploidization in crop improvement.

## Conclusion

The present study as the first to document 2*n* gamete occurrence in cultivated cymbidiums. Depending on cultivars, 2*n* male gamete formation frequencies varied from 0.15 to 4.03%. Hybrid cultivars, especially those interspecific ones have relatively higher frequencies of 2*n* male gamete formation. Hybridization using parents with higher 2*n* male gamete formation frequencies generally have a high probability to produce polyploid progenies. Five triploid and two tetraploid progenies were produced in this study. Triploid sterility is not an obstacle to ornamental plants as they are valued for the aesthetic appearance, not for seed production. Triploid plants can be propagated through tissue culture to increase plant numbers for commercial production. Characterization of two triploid plants showed that they exhibited improved ornamental value, i.e., rounder flowers with wider sepal, petals and lips. The sexual triploids had higher regeneration capacity during *in vitro* culture or displayed increased plant growth. Our results demonstrate that sexual polyploidization through unreduced gametes is a viable way for improving cymbidium.

## Data Availability Statement

The datasets generated for this study are available on request to the corresponding author.

## Author Contributions

Z-SZ and R-ZZ designed the study. JZ and G-HD performed determination of unreduced male gamete in parents, hybrid combination making, seedling production, and polyploid identification. S-YX and H-RG cultivated the hybrid and completed the effects of sexual polyploidization on micropropagation characteristics and ornamental traits. LX, R-ZZ, and Z-SZ carried out and analyzed the data. R-ZZ wrote a first draft of the manuscript that was further critically reviewed by JC, Z-SZ, LX, JZ, S-YX, H-RG, and G-HD.

## Conflict of Interest

The authors declare that the research was conducted in the absence of any commercial or financial relationships that could be construed as a potential conflict of interest.
