# Supplementary figures and images for: Unreduced Male Gamete Formation in Cymbidium and Its Use for Developing Sexual Polyploid Cultivars
Source: Front Plant Sci. 2020 May 15;11:558. doi: 10.3389/fpls.2020.00558 (PMC7243674; doi:10.3389/fpls.2020.00558)

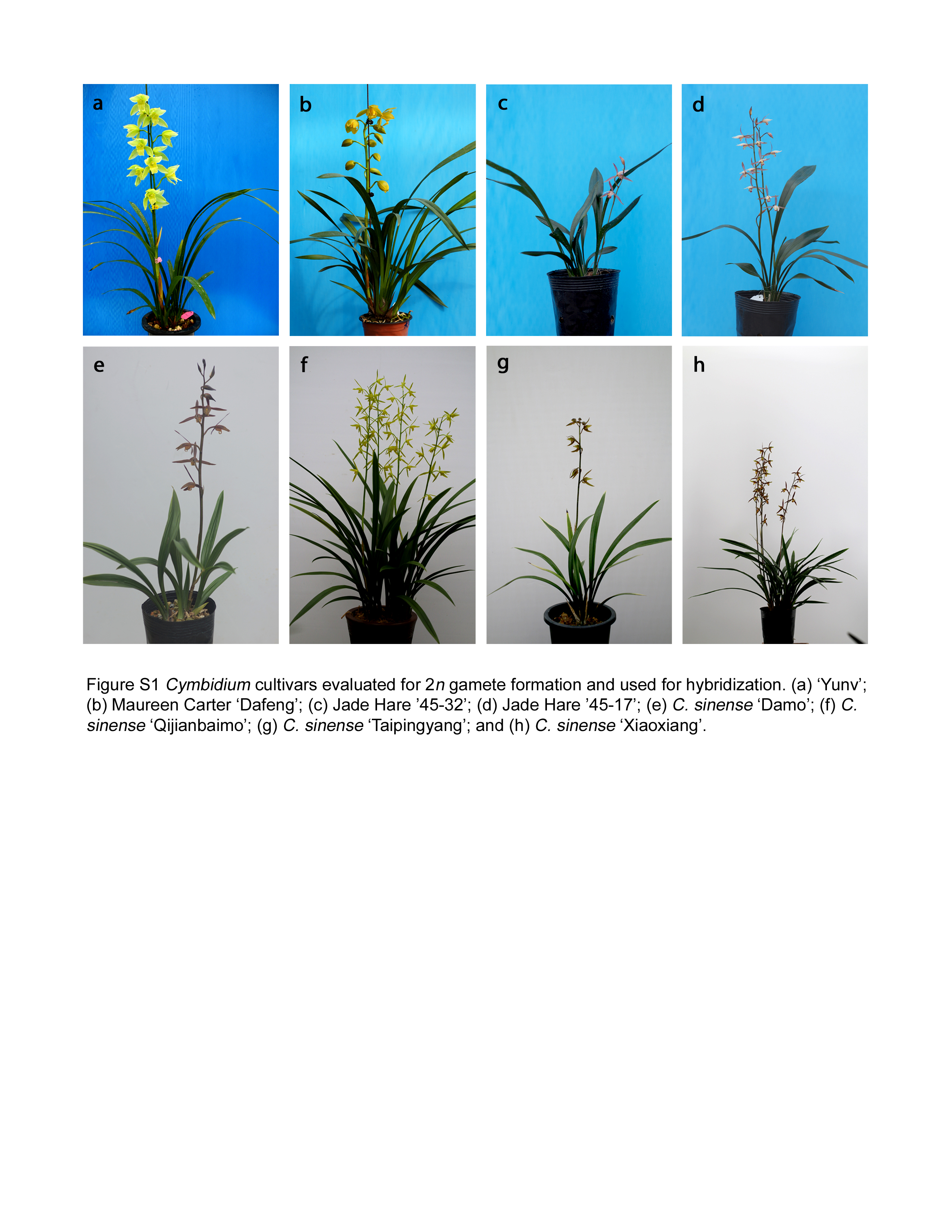

Supplement: Supplementary file 1 [file Image_1.JPEG]

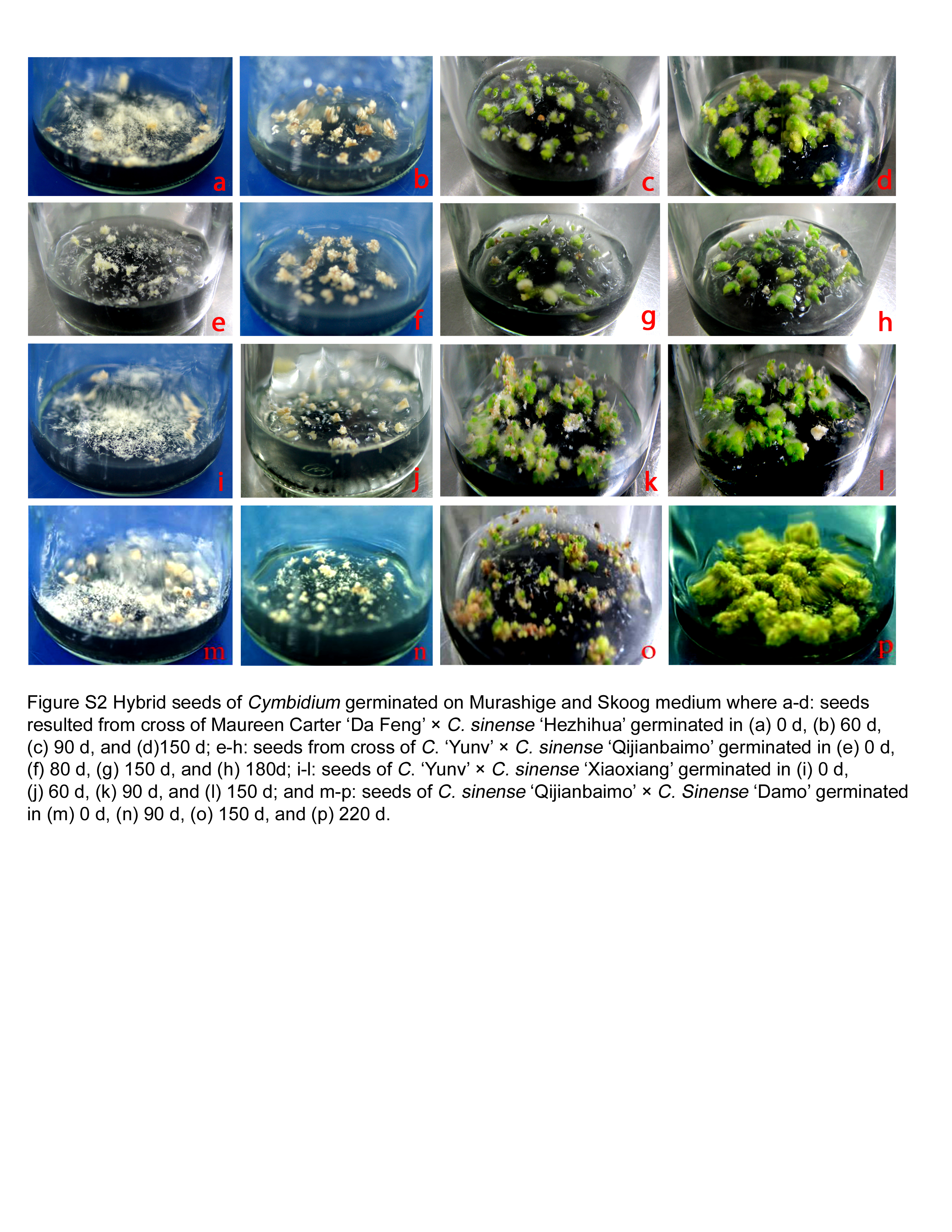

Supplement: Supplementary file 2 [file Image_2.JPEG]

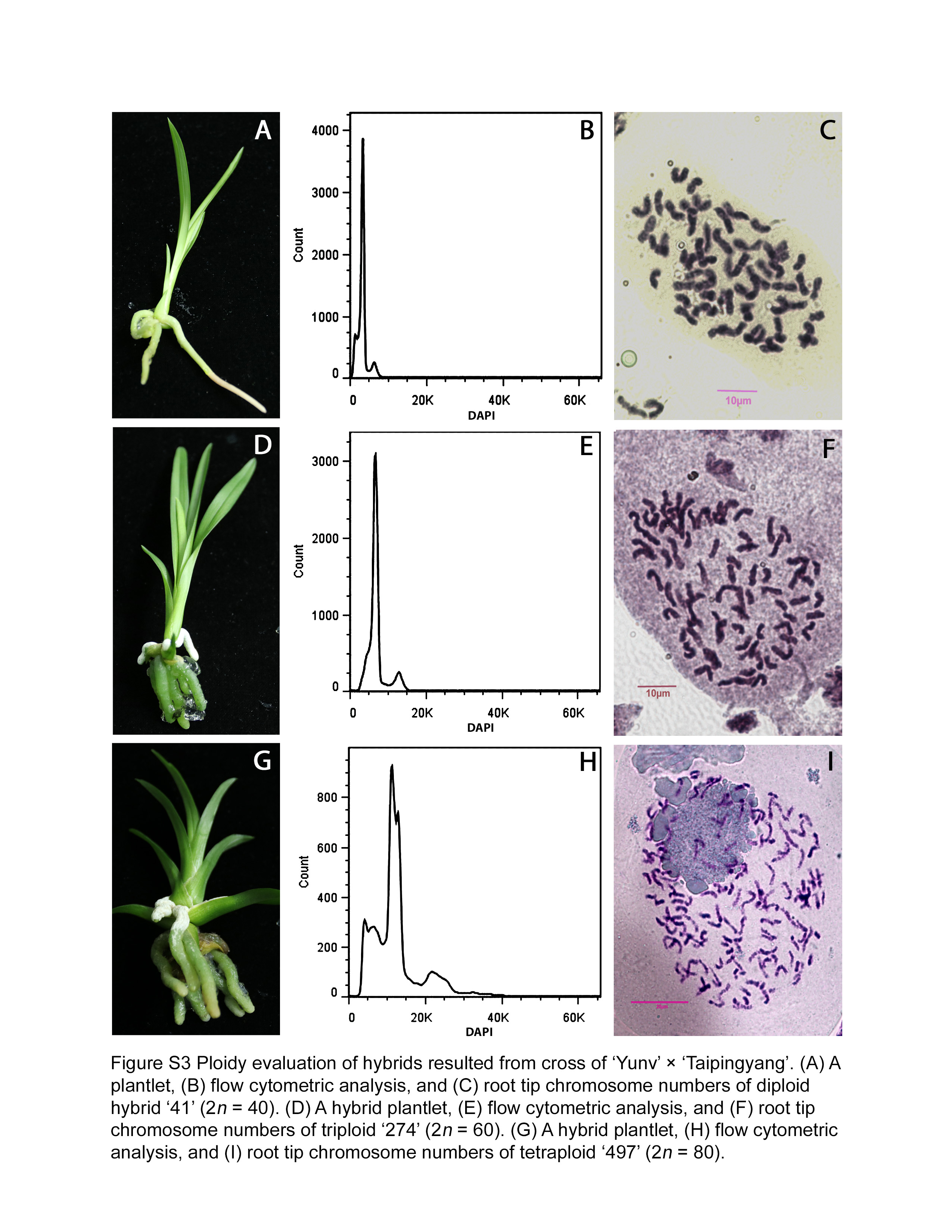

Supplement: Supplementary file 3 [file Image_3.jpg]

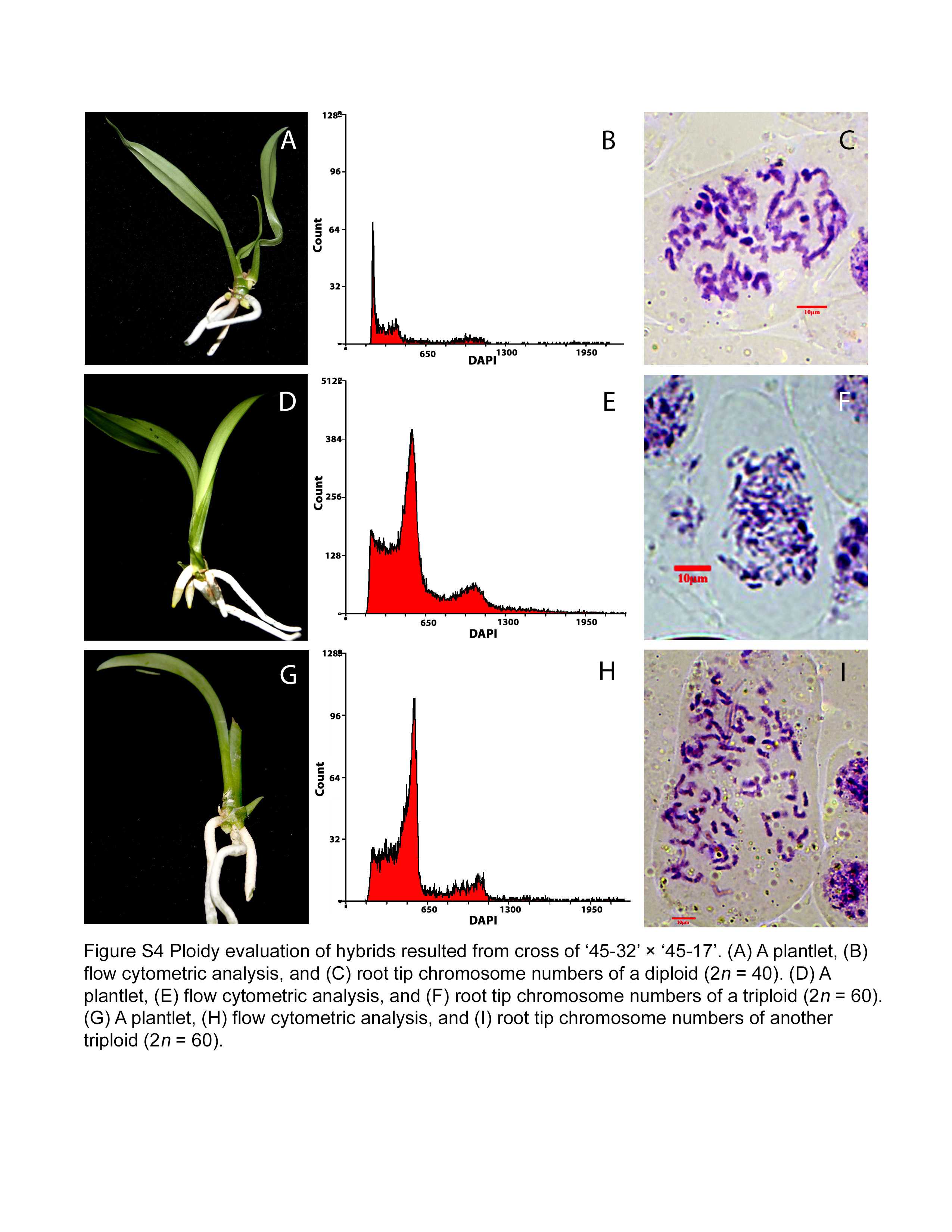

Supplement: Supplementary file 4 [file Image_4.jpg]

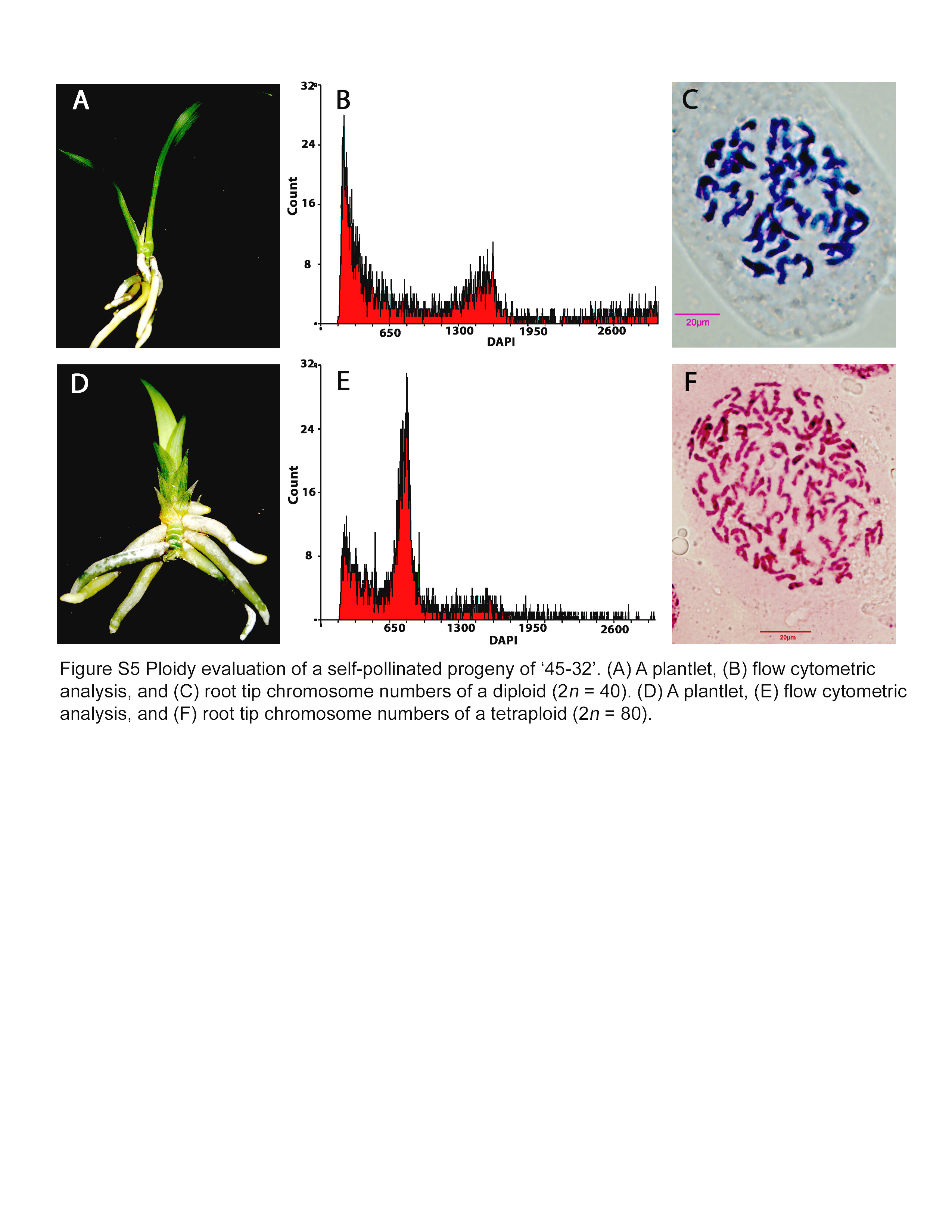

Supplement: Supplementary file 5 [file Image_5.jpg]
