# Supplementary material for: Unreduced Male Gamete Formation in Cymbidium and Its Use for Developing Sexual Polyploid Cultivars
Source: Front Plant Sci. 2020 May 15;11:558. doi: 10.3389/fpls.2020.00558 (PMC7243674; doi:10.3389/fpls.2020.00558)
Supplement: Supplementary file 6 [file Table_1.DOC]

**Table S1** Observed dyads and triads and 2*n* gamete occurrence frequencies in pollens of 10 *Cymbidium* cultivars during a three-year evaluation

| Cultivars | 2012 | | | | 2013 | | | | 2014 | | | |
| --- | --- | --- | --- | --- | --- | --- | --- | --- | --- | --- | --- | --- |
|  | No. of microspore | No. of dyads | No. of triads | Occurrence frequency of 2*n* male gamete (%) | No. of microspore | No. of dyads | No. of triads | Occurrence frequency of 2*n* male gamete (%) | No. of microspore | No. of dyads | No. of triads | Occurrence frequency of 2*n* male gamete (%) |
| *C. sinense* ‘Qijianbaimo’ | 705 | 0 | 25 | 0.89 | 496 | 0 | 14 | 0.71 | 432 | 0 | 7 | 0.41 |
| *C. sinense* ‘Damo’ | 455 | 0 | 3 | 0.17 | 374 | 0 | 7 | 0.47 | 219 | 0 | 2 | 0.23 |
| *C. sinense* ‘Hezhihua’ | 411 | 0 | 3 | 0.18 | 319 | 0 | 5 | 0.39 | 372 | 0 | 13 | 0.88 |
| *C. sinense* ‘Xiaoxiang’ | 460 | 0 | 3 | 0.16 | 499 | 0 | 2 | 0.10 | 540 | 0 | 4 | 0.19 |
| *C. sinense* ‘Taipingyang’ | 568 | 4 | 5 | 0.58 | 468 | 2 | 2 | 0.32 | 390 | 1 | 5 | 0.45 |
| *C.* ‘Yunv’ | 273 | 1 | 2 | 0.37 | 402 | 1 | 2 | 0.25 | 322 | 1 | 2 | 0.31 |
| *C.* Maureen Carter ‘Dafeng’ | 305 | 2 | 15 | 1.58 | 294 | 2 | 7 | 0.94 | 379 | 2 | 2 | 0.4 |
| *C.* Jade Hare ‘45-17’ | 927 | 56 | 81 | 5.49 | 865 | 37 | 67 | 4.25 | 543 | 10 | 30 | 2.36 |
| *C.* Jade Hare ‘45-32’ | 803 | 49 | 43 | 4.59 | 969 | 12 | 32 | 1.45 | 591 | 1 | 44 | 1.98 |
| *C. lancifolium* | 541 | 1 | 3 | 0.23 | 583 | 4 | 5 | 0.56 | 607 | 1 | 7 | 0.37 |
