# Supplementary material for: Unreduced Male Gamete Formation in Cymbidium and Its Use for Developing Sexual Polyploid Cultivars
Source: Front Plant Sci. 2020 May 15;11:558. doi: 10.3389/fpls.2020.00558 (PMC7243674; doi:10.3389/fpls.2020.00558)
Supplement: Supplementary file 7 [file Table_2.DOCX]

**TABLE S2** Hybridization combination and hybrid seed germination of *Cymbidium*

| Cross combination | Cross success rate (%) | Fruit developmental period (days) | Seed germination duration (days) | Seed germination frequency (%) |
| --- | --- | --- | --- | --- |
| ‘Dafeng’ × ‘Hezhihua’ | 100 | 310 | 150 | 90.0 |
| ‘Yunv’ × ‘Qijianbaimo’ | 100 | 214 | 150 | 70.0 |
| ‘Qijianbaimo’ × ‘Damo’ | 100 | 245 | 250 | 90.0 |
| ‘Yunv’ × ‘Xiaoxiang’ | 100 | 214 | 150 | 70.0 |
| ‘Yunv’ × ‘Taipingyang’ | 100 | 210 | 150 | 90.0 |
| ‘45-32’ × ‘45-17’ | 100 | 238 | 152 | 30.0 |
| ‘45-32’ × ‘45-32’ | 100 | 238 | 152 | 30.0 |

Note: The cross-success rate was calculated based on the number of mature capsules obtained divided by the number of flowers pollinated.

Seed germination frequency (%) = (no. of rhizomes/estimated number of seeds sown per culture vessel) x 100. Depending on the availability of hybrid seeds, approximately 50 to 300 seeds were sown onto half-strength MS medium per culture vessel.
